# Chemical Components of Volatile Oils and Hydrosols Derived from Different Plant Organs of Three *Scutellaria* L. Species

**DOI:** 10.3390/plants15142239

**Published:** 2026-07-22

**Authors:** Tatiana Rodideal, Nina Ciocârlan, Lăcrămioara Carmen Ivănescu, Mădălina-Elena Frunzete, Maria-Magdalena Zamfirache, Violeta Alexandra Ion, Liliana Bădulescu

**Affiliations:** 1 Faculty of Biology, “Alexandru Ioan Cuza” University, Bd. Carol I, Nr. 20 A, 700505 Iasi, Romania; tatiana.rodideal@yahoo.com (T.R.); f.madalinaelena@yahoo.com (M.-E.F.); magda@uaic.ro (M.-M.Z.); 2 National Botanical Garden (Institute) “Al. Ciubotaru”, Moldova State University, 18 Padurii Str., 2002 Chisinau, Moldova; nina.g.ciocarlan@gmail.com; 3 Research Center for Studies of Food Quality and Agricultural Products, University of Agronomic Sciences and Veterinary Medicine of Bucharest, 59 Mărăşti Blvd., District 1, 011464 Bucharest, Romania; violeta.ion@qlab.usamv.ro (V.A.I.); liliana.badulescu@qlab.usamv.ro (L.B.); 4 Faculty of Horticulture, University of Agronomic Sciences and Veterinary Medicine of Bucharest, 59 Mărăști Blvd., District 1, 011464 Bucharest, Romania

**Keywords:** *Scutellaria*, *altissima*, *baicalensis*, *supina*, volatile oils, hydrosols, neoclevenger, GC-MS, *n*-hexane, DiBP

## Abstract

The genus *Scutellaria* L. occupies an isolated position within the family Lamiaceae, whereas the aerial organs of many species remain largely unexplored despite their potential as sources of bioactive metabolites. This study provides the first comprehensive comparison of 20 volatile oils and their corresponding 20 hydrosols obtained by hydrodistillation from different organs of three *Scutellaria* species cultivated at NBGI (Republic of Moldova). GC–MS analysis identified eleven classes of secondary metabolites. *S. altissima* L. was characterized by linalool as the principal constituent in volatile oils (20.24–29.96%) and hydrosols (55.36–61.72%). *S. baicalensis* Georgi produced sesquiterpene-rich volatile oils primarily composed of β-caryophyllene (23.44–37.99%) and germacrene D (6.83–22.42%), whereas its hydrosols contained high relative abundances of 1-octen-3-ol (24.54–59.32%). The volatile oils of *S. supina* L. were characterized by high relative abundances of germacrene D (up to 33.16%), β-caryophyllene (up to 21.72%), linalool (up to 21.95%), and spathulenol (up to 11.36%). In contrast, hydrosols contained high relative abundances of 1-octen-3-ol (17.39–71.10%) and linalool (5.86–45.49%). Hierarchical cluster analysis distinguished volatile oils from hydrosols and revealed species-specific volatile profiles, with organ-related variation being primarily quantitative. These results underscore the potential of aerial organs and hydrosols for future investigations.

## 1. Introduction

The genus *Scutellaria* L. occupies an isolated position within the family Lamiaceae [1] and comprises approximately 400–500 species [2]. Species within this genus are primarily distributed across the grasslands, open forests, and scrublands of the Northern Hemisphere, particularly in temperate regions [3]. Within the genus, species classification is mainly based on morphological characteristics [4,5], although taxonomic approaches incorporating biochemical studies have also proven valuable [6]. *Scutellaria baicalensis* Georgi is the most extensively studied taxon, with its pharmacological potential evaluated both *in vivo* and *in vitro*, including animal models [7,8] and pilot clinical studies involving human subjects [9,10]. Traditionally, the underground organs of *Scutellaria* plants are used, whereas the aerial organs are often considered agricultural waste and discarded [11]. Recent research, however, has highlighted the potential applications of these aerial organs in aquaculture [12,13], the poultry industry [14,15], the swine industry [16,17], and the ruminant industry [18,19] due to their antimicrobial, antiviral, and immunostimulatory properties and beneficial effects on the intestinal microbiota. Compared with secondary metabolites of the flavonoid class [20], volatile oils of *Scutellaria* have received less attention. This limited focus is likely due to earlier reports suggesting that, relative to other Lamiaceae, *Scutellaria* species are not aromatic and contain less volatile oil [4,21]. Hydrosols have also been understudied, as they are often regarded as by-products of the distillation process and subsequently discarded. Nevertheless, recent studies have demonstrated that hydrosols may possess antimicrobial, antioxidant, and other biological properties, attributed to active compounds dissolved in the aqueous phase [22]. Comprehensive studies examining volatile oils and hydrosols from different plant organs (stems, leaves, flowers, and fruits) of the same *Scutellaria* species, as well as comparative analyses of volatile oils and their corresponding hydrosols, remain scarce. Furthermore, no previous studies have investigated the composition of the volatile oils and hydrosols of *Scutellaria* taxa cultivated at the National Botanical Garden (Institute) “Al. Ciubotaru” (NBGI) in the Republic of Moldova. The NBGI collection also includes *S. supina* L., a critically endangered species prevalent on the Podolia Plateau (Hrușca) and listed in the Red Book of the Republic of Moldova [23,24]. A survey of the available literature indicates that the volatile oil composition of *S. supina* has not been previously reported. Collectively, these knowledge gaps provide the rationale for the present study and demonstrate its novelty.

The objective of this study was to characterize, using gas chromatography–mass spectrometry (GC-MS), the volatile oils and hydrosols obtained by hydrodistillation from the stems, leaves, flowers, and fruits of *S. altissima*, *S. baicalensis*, and *S. supina* cultivated at the NBGI during 2022–2023. Additionally, samples of *S. supina* from Dr. Pavel Pînzaru collection (2022) were used, together with volatile oils and hydrosols obtained from fresh leaves and stems of *S. baicalensis* collected in 2023.

We hypothesized that the concentrations of compounds present in *Scutellaria* taxa would vary among the volatile oils and hydrosols, species, and across distinct plant organs.

## 2. Materials and Methods

### 2.1. Plant Material

The biological material consisted of vegetative organs (stems and leaves) and reproductive organs (flowers and fruits) collected during the flowering–fruiting phenophase from *S. altissima*, *S. baicalensis*, and *S. supina* (harvested in 2023), which were cultivated in the experimental field of the National Botanical Garden (Institute) “Alexandru Ciubotaru” in Chisinau, Republic of Moldova. Collection and harvesting were conducted in accordance with Access Document No. SD 337/2.08.2022. The plant material from *S. supina* collected in 2022 was obtained from the personal collection of Doctor of Biological Sciences Pavel Pînzaru. Representative specimens of each taxon were deposited in the Herbarium of the Faculty of Biology, Alexandru Ioan Cuza University of Iasi, Romania. Inventory numbers (vouchers) were assigned as follows: *S. altissima*: I 207159; *S. baicalensis*: I 207016; *S. supina*: I 207158.

#### 2.1.1. Preparation of Plant Material

Plant material was dried at room temperature. After drying, vegetative and reproductive organs were separated and stored individually in paper bags. From *S. baicalensis* specimens collected in 2023, vegetative organs (leaves and stems) were separated from fresh material to assess differences in chemical composition between fresh and dried samples. Before steam distillation, dried plant organs were ground into a fine powder [25], whereas fresh organs were cut into small fragments [26,27]. The amount of plant material used in each hydrodistillation was standardized at 100 g for all stem and leaf samples. For flowers and fruits, the limited availability of material required that all available biomass from collected plants be hydrodistilled. This methodology was adopted due to the small scale of field collections and the conservation priorities of NBGI, which focus on seed harvesting, introduction, and maintenance of *Scutellaria* taxa in *ex situ* collections [23]. To preserve reproductive organs, destructive sampling was minimized (Appendix A).

#### 2.1.2. Steam Distillation of Plant Material

Steam distillation was conducted using a Neo-Clevenger-type hydrodistillation apparatus (Energo-Metr SRL, Odorheiu Secuiesc, Harghita County, Romania) for 3 h and 30 min. The duration was recorded from the moment the mixture in the flask reached its boiling point [28,29]. A total of 20 volatile oil samples and 20 hydrosol samples were obtained. For each plant species and organ, one replicate was hydrodistilled.

#### 2.1.3. Collection and Storage of Volatile Oil and Hydrosol Samples

At the end of the distillation period, after the refrigerant had cooled completely, a portion of the hydrosol was collected into sterile 15 mL Falcon tubes. The volatile oil was collected in an amber vial for chromatographic analysis using *n*-hexane [30,31]. The samples were sealed with Parafilm and stored at −18 °C until analysis. Extraction yields (%) were not calculated due to the limited availability of plant material and the extremely low amount of essential oil obtained.

### 2.2. Analysis of the Biochemical Composition of Volatile Oils and Hydrosols Using GC-MS

#### 2.2.1. Preparation of Volatile Oil Samples for GC-MS Analysis

For sample preparation, 100 µL of volatile oil was mixed with 900 µL of *n*-hexane, yielding a final dilution of 1:10 (*v*/*v*) for each sample.

#### 2.2.2. Preparation of Hydrosol Samples for GC-MS Analysis

Hydrosol samples were prepared as follows: one milliliter of *n*-hexane was added to the hydrosol volumes specified in Appendix A Table A1. The mixtures were vortexed for 30 s and then allowed to settle until phase separation occurred. Subsequently, 0.25 mL of the organic phase was collected and transferred to vials for GC-MS analysis. Each volatile oil and hydrosol sample was analyzed by GC-MS with a single analytical replicate.

#### 2.2.3. Analytical Procedures for Oil and Hydrosol Samples

Sample analysis was conducted using an Agilent Series gas chromatography–mass spectrometry (GC-MS) system comprising a GC 7890B gas chromatograph and a 5977A MS system with a GS Sampler 80 injector (Agilent Technologies, Santa Clara, CA, USA). The procedure used an HP-5MS ultra-inert column (30 m × 0.25 mm × 0.25 µm film thickness; Agilent Technologies, Santa Clara, CA, USA). The column oven temperature program included an initial hold at 50 °C for 8 min, followed by a linear increase to 280 °C at 4 °C/min. Helium served as the carrier gas at a flow rate of 1 mL/min. The injection volume was 3 µL, with a split ratio of 50:1. The ionization energy was set to 70 eV, and mass spectra were recorded in the 50–550 *m*/*z* range. A homologous series of *n*-alkanes (decane, dodecane, tetradecane, hexadecane, octadecane, eicosane, docosane, and tetracosane) at 50 µg/mL was analyzed under the same chromatographic conditions as the samples and used to calculate linear retention indices (LRI) for compound identification. All GC-MS analyses were performed under identical conditions. Data acquisition and processing were carried out using Agilent MSD Productivity ChemStation for GC and GC/MS Systems Data Analysis software (version F.01.00.1903, Agilent Technologies, Santa Clara, CA, USA).

#### 2.2.4. Identification and Quantification of Secondary Metabolites

Secondary metabolites were identified based on their mass spectra and linear retention indices (LRI). Qualitative identification involved comparing experimentally obtained mass spectra with reference spectra from the National Institute of Standards and Technology (NIST) Mass Spectral Library (NIST/EPA/NIH Mass Spectral Library, NIST 14. L), using Reverse Match (R Match) scores and comparing the calculated LRI values with published literature data [32,33]. The relative abundance of each compound was calculated by normalizing its peak area to the total chromatographic peak area and was expressed as a percentage of the total peak area [34,35].

#### 2.2.5. Retention Index Calculation

Considering the use of the temperature-programming regime, the linear retention index (LRI) was calculated using the equation proposed by [36] using Microsoft Excel.

=100*((tR(x) − tR(y))/(tR(z) − tR(y)) + 100*y(1)

where tR(x) is the retention time of the compound of interest; tR(y) is the retention time of the *n*-alkane eluting immediately before the compound of interest; tR(z) is the retention time of the *n*-alkane eluting immediately after the compound of interest; y is the number of carbon atoms in the *n*-alkane eluting immediately before the compound of interest.

### 2.3. Hierarchical Cluster Analysis

Hierarchical cluster analysis (HCA) was performed using the Ward agglomerative algorithm and Euclidean distance based on the standardized data matrix of relative abundance for the identified compounds [37,38]. The Ward method has been widely applied in the chemometric analysis of volatile oils and other phytochemical datasets [39,40]. Dendrograms were generated to visualize the chemical similarity among the samples analyzed. All multivariate analyses were performed using PAST software [41].

## 3. Results

### 3.1. GC-MS Characterization of Volatile Oil and Hydrosol Derived from S. altissima

Four secondary metabolites, classified as oxygenated monoterpenes (linalool, α-terpineol, nerol, and geraniol), were identified in all oil samples and their corresponding hydrosols, with higher relative abundances in the hydrosols. For example, linalool had a relative abundance of 24.71% in the volatile oil from stems, compared with 59.21% in the corresponding hydrosol. Benzaldehyde and eugenol were identified exclusively in all hydrosol samples, with relative abundances ranging from 0.24% to 2.10%. In contrast, seven compounds (β-ionone, α-patchoulene, E-nerolidol, n-hexyl salicylate, hexa-hydrofarnesyl acetone, palmitic acid, and phytol) were identified only in volatile oil samples, and their relative abundances varied considerably among samples. For instance, the relative abundance of E-nerolidol was highest in stems (1.01%) and leaves (2.47%), while that of palmitic acid was highest in flowers (15.72%) and fruits (20.56%). D-limonene was identified only in volatile oils from stems (0.10%) and leaves (0.12%), while farnesyl acetone was absent from volatile oils and hydrosols derived from stems (Appendix A—Table A2).

**Figure 1:**
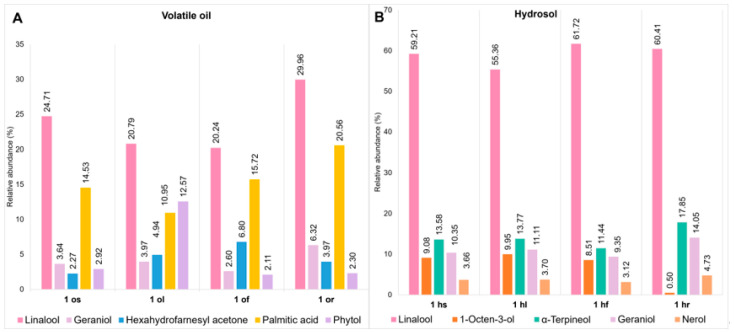
Five major metabolites with the highest relative abundance in volatile oil (**A**) and hydrosol (**B**) samples of *S. altissima* (1) harvested in 2023; Abbreviations: o = volatile oil; h = hydrosol; l = leaves; s = stems; f = flowers; r = fruits.

Volatile oil and hydrosol samples exhibited high relative abundances of linalool (20.70–29.96% in oils; 55.36–61.72% in hydrosols) and geraniol (2.60–6.32% in oils; 9.35–14.05% in hydrosols). Volatile oils exhibited higher relative abundances of palmitic acid, phytol, and hexahydrofarnesyl acetone. In contrast, hydrosol samples showed higher relative abundances of α-terpineol, geraniol, and nerol (Figure 1).

Alcohols (0.50–15.97%), ketones (0.26–7.85%), and oxygenated monoterpenes (26.10–97.04%) were identified in all investigated samples; however, their relative abundances varied widely. For instance, the relative abundance of oxygenated monoterpenes in volatile oil from stems was 32.31%, while that from leaves was 29.32%. In the corresponding hydrosols, this class of compounds reached 86.79% and 83.94%, respectively. Certain compound classes, such as acids (13.41–21.68%), sesquiterpene hydrocarbons (0.43–0.80%), and oxygenated sesquiterpenes (0.79–2.47%), were identified exclusively in volatile oils. Monoterpene hydrocarbons were present only in volatile oils from stems (0.10%) and leaves (0.12%). In contrast, aldehydes (0.24–2.10%) and phenols (0.28–0.62%) were identified exclusively in hydrosols (Table 1).

### 3.2. GC-MS Characterization of Volatile Oil and Hydrosol Derived from S. baicalensis

The analyzed samples of *S. baicalensis* collected in 2022 contained a common set of eight compounds: 1-octen-3-ol, 3-octanol, linalool, germacrene D, γ-cadinene, spathulenol, caryophyllene oxide, and alloaromadendrene oxide-(1). The compounds α-cubebene, E-nerolidol, cubenol, and a compound with the chemical formula C_15_H_24_O were identified exclusively in all oil samples, while the compounds benzaldehyde and eugenol were identified only in hydrosols obtained from the four plant organs. Carvone was identified exclusively in the hydrosol derived from stems (2.91%) and fruits (0.46%), γ-elemene and humulene epoxide II were present only in the volatile oils extracted from leaves, flowers, and fruits, whereas aromandendrene was identified only in samples from stems, leaves, and flowers. Certain compounds were identified in only one sample; for example, α-bergamotene (3.40%) was found only in the volatile oil from stems, β-ionone (0.61%) only in stem hydrosol, and 1-undecanol in both the volatile oil (3.21%) and hydrosol (0.95%) from stems (Appendix A—Table A3).

In the plant material collected in 2023, germacrene D was the only compound identified in all analyzed samples, with relative abundances significantly higher in volatile oils (10.00–22.42%) than in the corresponding hydrosols (0.43–9.37%). The compounds tridecane and eugenol were identified exclusively in all hydrosol samples, whereas seven compounds (γ-elemene, α-cubebene, β-copaene, (E.)-β-farmesene, α-humulene, bicyclogermacrene, and farnesene) were identified only in oil samples. Volatile oil samples exhibited high relative abundances of β-bourbonene, germacrene D, and caryophyllene oxide. Some compounds were identified exclusively in specific samples; for example, benzaldehyde was identified only in hydrosols from fruits, fresh stems, and leaves, while phytol was detected solely in the oil from dried leaves and fruits. β-elemene, α-muurolene, and E-nerolidol were identified in the volatile oil from dried stems but were absent from volatile oils from fresh stems (Appendix A—Table A4).

**Figure 2:**
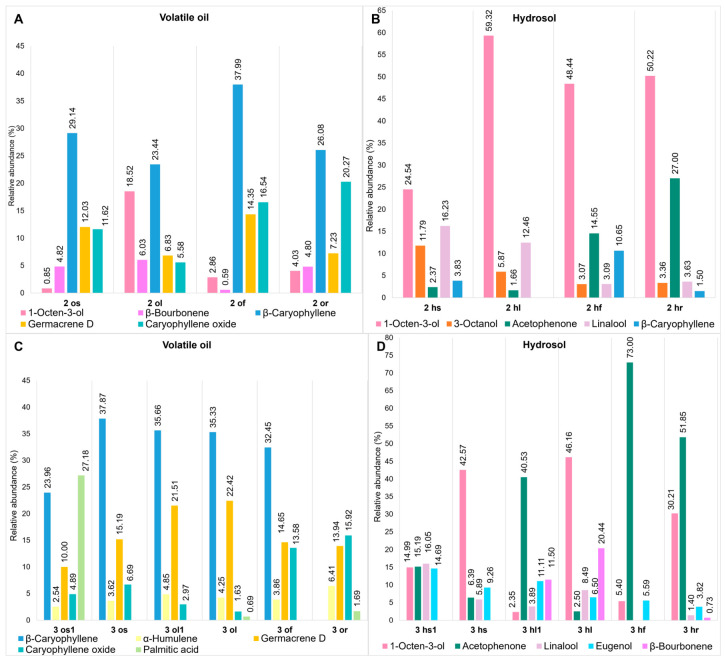
Five major metabolites with the highest relative abundance in volatile oil (**A**,**C**) and hydrosol (**B**,**D**) samples of *S. baicalensis* harvested in 2022 (2) and 2023 (3). Abbreviations: o = volatile oil; h = hydrosol; l = leaves; s = stems; f = flowers; r = fruits; s 1 = fresh stems; l 1 = fresh leaves.

Across both years of *S. baicalensis* collection, benzaldehyde, tridecane, and eugenol were identified exclusively in hydrosol samples. In contrast, E-nerolidol, cubenol, and γ-elemene were identified solely in certain oil samples. For plants collected in 2023, α-copaene, β-caryophyllene, and β-copaene were identified exclusively in some oil samples, whereas in 2022, these compounds were also present in hydrosols. Except for carvone, α-bergamotene, 1-undecanol, and β-ionone, which appeared only in specific 2022 samples, all other compounds were common to plants collected in both years. Volatile oil samples from plants collected in two consecutive years exhibited high relative abundance of β-caryophyllene, germacrene D, and caryophyllene oxide. Except for caryophyllene oxide, the relative abundances of these compounds were considerably higher in samples from plants collected in 2023. Additionally, plants collected in 2022 contained high relative abundances of 1-octen-3-ol and β-bourbonene, whereas plants collected in 2023 were distinguished by the presence of α-humulene and palmitic acid (Figure 2A,C). The relative abundances of β-caryophyllene, germacrene D, and α-humulene seem to remain relatively constant regardless of the processing status of the plant material (dried and fresh leaves). Hydrosol samples obtained from plants collected in 2022 showed higher relative abundances of 1-octen-3-ol, 3-octanol, and β-caryophyllene, whereas plants collected in 2023 showed higher relative abundances of acetophenone, eugenol, and β-bourbonene. Samples obtained from the flowers and fruits showed considerably higher relative abundances of acetophenone, particularly in plants collected in 2023 (flowers: 73.00; fruits: 51.85%) compared to those collected in 2022 (flowers: 14.55; fruits: 27.00%). Stem hydrosol was characterized by higher relative abundances of linalool, whereas hydrosol from dried leaves was characterized by the presence of 1-octen-3-ol (leaves collected in 2022: 59.32%; leaves collected in 2023: 46.16%). The relative abundance of this compound was considerably lower in the hydrosol obtained from fresh leaves (2.35%) (Figure 2B,D).

In all samples obtained from plants collected in 2022 (Table 2), compounds belonging to the alcohol, oxygenated monoterpene, oxygenated sesquiterpene, and sesquiterpene hydrocarbon classes were identified, whereas in all samples obtained from plants collected in 2023 (Table 3), only compounds belonging to the ketone and sesquiterpene hydrocarbon classes were identified. Regardless of the year of collection, compounds belonging to the aldehyde, alkane, and phenol classes were identified exclusively in hydrosol samples. The relative abundances of secondary metabolite classes varied considerably between volatile oil and hydrosol samples and among plant organs. For example, the relative abundance of alcohols in the volatile oil obtained from stems collected in 2022 was 4.83%, whereas it reached 37.96% in the corresponding hydrosol. A similar pattern was observed for the sample obtained from dried stems collected in 2023, with relative abundances of 1.95% in the volatile oil and 57.13% in the corresponding hydrosol. Volatile oils were characterized by higher relative abundances of sesquiterpene hydrocarbons and oxygenated sesquiterpenes, whereas hydrosols exhibited higher relative abundances of alcohols, oxygenated monoterpenes, and ketones. Compounds belonging to the acid class had a relative abundance of 27.18% in the volatile oil obtained from fresh stems, were absent from the volatile oil obtained from dried stems, and were present at a lower relative abundance (1.13%) in the corresponding hydrosol.

### 3.3. GC-MS Characterization of Volatile Oil and Hydrosol Derived from S. supina

**Figure 3:**
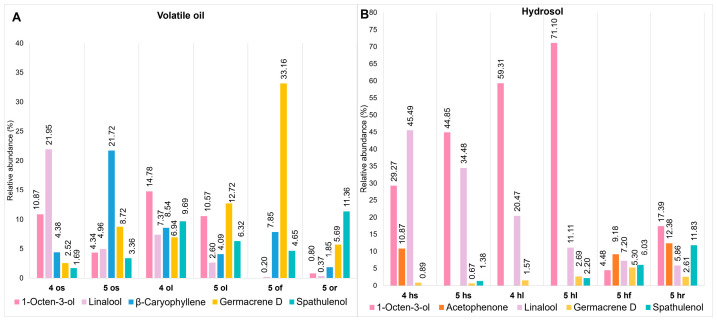
Five major metabolites with the highest relative abundance in volatile oil (**A**) and hydrosol (**B**) samples of *S. supina* harvested in 2022 (4) and 2023 (5); Abbreviations: o = volatile oil; h = hydrosol; l = leaves; s = stems; f = flowers; r = fruits.

Linalool and germacrene D were identified in all analyzed samples, with the highest relative abundances of linalool observed in hydrosol samples (5.86–45.49%), whereas germacrene D reached its highest relative abundance in volatile oil samples (2.52–33.16%). Tridecane was identified exclusively in all hydrosol samples; in contrast, β-copaene, (E)-β-famesene, and aromandendrene were identified only in volatile oil samples, with their highest relative abundances observed in the volatile oil extracted from leaves collected in 2022. Carvone was identified only in hydrosol samples obtained from stems (2.14%) and leaves (1.21%) collected in 2022. Conversely, γ-elemene, β-eiemene, 2-butanone, 4-(2,2-dimethyl-6-methylenecyclohexyl), and β-cubebene were identified exclusively in plants collected in 2023. Aloaromadendren oxide-(1) was identified in all hydrosol samples obtained from plants collected in 2023 but was absent from those obtained from plants collected in 2022. The compounds γ-elemene (1.60%), β-cubebene (4.18%), α-muurolene (2.07%) and germacrene D (33.16%) reached their highest relative abundances (1.60%) in the volatile oil from flowers collected in 2023. Caryophyllene oxide (5.42%) and humulene epoxide II (5.24%) exhibited their highest relative abundances in the volatile oil from leaves collected in 2022, whereas palmitic acid (19.10%) and 1-undecanol (3.45%) reached their highest relative abundances in stems collected in the same year (Appendix A—Table A5). The volatile oil samples were characterized by higher relative abundances of β-caryophyllene, germacrene D, and spathulenol, whereas the hydrosols were characterized by higher relative abundances of 1-octen-3-ol, linalool, and acetophenone. The volatile oil obtained from stems and leaves collected in 2022 showed higher relative abundances of 1-octen-3-ol and linalool than that obtained from plants collected in 2023. The volatile oil obtained from the flowers showed the highest relative abundance of germacrene D (33.16%), whereas the volatile oil from the fruits showed the highest relative abundance of spathulenol (11.36%). In the hydrosol samples obtained from leaves collected in 2022 (44.85%) and 2023 (59.31%), the compound 1-octen-3-ol showed the highest relative abundance, compared to the samples obtained from other organs (4.48–44.85%). As with volatile oil, the hydrosol obtained from the fruits showed the highest relative abundances of spathulenol (11.83%) and acetophenone (12.38%) (Figure 3).

Compounds belonging to the alcohol, oxygenated monoterpene, and sesquiterpene hydrocarbon classes were identified in all analyzed samples. Except for sesquiterpene hydrocarbons, alcohols and oxygenated monoterpenes were present at higher relative abundances in hydrosol samples. Alkanes, with relative abundances ranging from 0.68 to 2.40%, were identified in all hydrosol samples. Compounds belonging to the acid class were identified in the volatile oil obtained from stems collected in 2022 (19.10%) and in some hydrosol samples at substantially lower relative abundances (0.70–2.41%). In volatile oil samples, oxygenated monoterpenes exhibited higher relative abundances in stems collected in 2022 (21.95%) than in stems collected in 2023 (4.96%). Likewise, oxygenated sesquiterpenes showed higher relative abundances in volatile oil from leaves collected in 2022 (23.45%) than in that obtained from leaves collected in 2023 (9.24%). The volatile oil (61.88%) and the corresponding hydrosol (14.92%) obtained from flowers exhibited high relative abundances of sesquiterpene hydrocarbons. Alcohols and oxygenated monoterpenes also exhibited substantially higher relative abundances in hydrosol samples obtained from plant stems and leaves (Table 4).

### 3.4. Comparative Analysis of Samples, Including Hierarchical Cluster Analysis

A comparative analysis of the chemical composition of volatile oils and hydrosols from three *Scutellaria* taxa collected in 2022 and 2023 revealed a shared chemical core of nine secondary metabolites: 1-octen-3-ol, acetophenone, linalool, α-terpineol, geraniol, eugenol, E-nerolidol, hexahydrofarnesyl acetone, and palmitic acid. Some compounds were common to all three taxa, whereas others were unique to specific taxa. For example, D-limonene, nerol, α-patchoulene, n-hexyl salicylate, myristic acid, and farnesyl acetone were identified exclusively in *S. altissima*. In contrast, α-(Z,E)-farnesene, bicyclogermacrene, and cubenol were identified only in *S. baicalensis*, while 2-methoxy-4-vinylphenol was unique to *S. supina*. Nevertheless, in the absence of a comprehensive, systematic evaluation with broader comparative analyses across multiple populations and related species, these compounds should not be interpreted as chemotaxonomic markers for the taxa under investigation. Certain compounds were exclusive to either hydrosol or volatile oil samples. Additionally, some compounds exhibited markedly higher relative abundances in hydrosols; for example, the relative abundance of linalool was 20.24% in the volatile oil and 61.72% in the hydrosol of *S. altissima* flowers. The relative abundance of acetophenone was 0.84% in the volatile oil obtained from fruits of *S. baicalensis* collected in 2022, whereas it reached 27% in the corresponding hydrosol.

**Figure 4:**
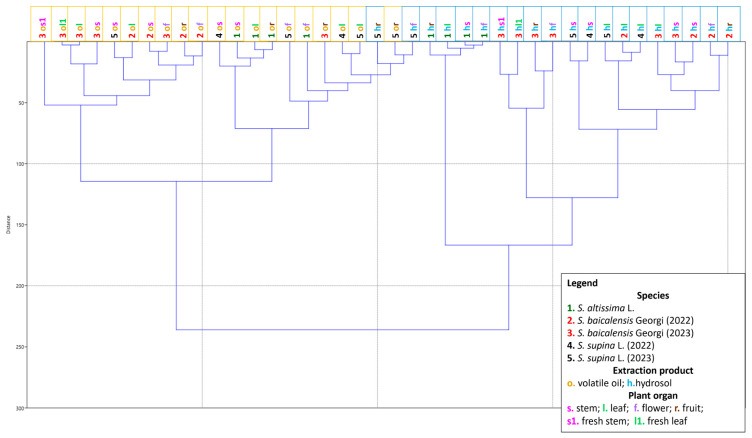
Hierarchical cluster analysis (HCA) of volatile oils and hydrosols from *Scutellaria* species.

Several secondary metabolites differed in their occurrences between *S. baicalensis* samples collected at the same site in 2022 and 2023. Carvone, a-bergamotene, 1-undecanol, and ß- ionone were identified only in the 2022 samples. In *S. supina*, carvone and (E)-ß-farnesene were identified exclusively in 2022 samples, while y-elemene, ß-elemene, 2-butanone, 4-(2,2-dimethyl-6-methylenecyclohexyl)-, and alloaromadendrene oxide-(1) were identified only in 2023 samples. The analysis of the three taxa revealed the presence of compounds from eleven classes of secondary metabolites (acids, alcohols, aldehydes, esters, alkanes, ketones, monoterpene hydrocarbons, phenols, sesquiterpene hydrocarbons, oxygenated monoterpenes and oxygenated sesquiterpenes). Esters and monoterpene hydrocarbons were identified exclusively in *S. altissima*. Aldehydes were identified in both *S. altissima* and *S. baicalensis*, while alkanes were identified in *S. supina* and *S. baicalensis*. The distribution of these compound classes differed considerably between volatile oils and hydrosols. Alkanes and aldehydes were observed only in hydrosols. Except for *S. supina*, phenols were also identified exclusively in hydrosols. Hierarchical cluster analysis (HCA) using Ward’s method and Euclidean distance separated the samples into two principal clusters based on extraction products. All volatile oil samples grouped together, while hydrosol samples formed a distinct second cluster. This pattern indicates that the type of hydrodistillation product is the primary source of chemical variation. Within each main cluster, samples were generally grouped by *Scutellaria* species, reflecting species-specific chemical profiles. Plant organs (stem, leaf, flower, and fruit) formed closely related subclusters within each species, with some overlap among organs. This overlap suggests that qualitative chemical compositions were largely similar, with differences primarily in the relative abundance of the constituents. Fresh leaf samples of *S. baicalensis* collected in 2023 clustered closely with the corresponding dried leaf, indicating only minor compositional changes due to drying. Samples collected in 2022 and 2023 were not clearly separated, suggesting that harvest year had a smaller influence on volatile composition than the extraction product or species (Figure 4).

## 4. Discussion

### 4.1. Comparative Analysis of Scutellaria altissima, S. baicalensis and S. supina

The production and accumulation of volatile oils show a non-homogeneous phylogenetic distribution associated with particular botanical families [42]. While certain compounds found in volatile oils are widespread across numerous plant species [33], others exhibit a limited distribution, occurring primarily within phylogenetically related groups [43]. The findings of the present study are not directly comparable to those reported previously due to differences in the plant material used for volatile oil extraction. In the current investigation, volatile oils and hydrosols were analyzed separately for each plant organ, whereas previous studies have generally examined either the entire aerial parts or a single plant organ. The chemical composition of the volatile oil of *S. altissima* has been reported in only two studies [44,45], while that of *S. baicalensis* has been described in only three studies [46,47,48]. To the best of our knowledge, no published studies have investigated the chemical composition of volatile oils obtained from *S. supina*. Likewise, only one study has reported the chemical composition of hydrosols from some *Scutellaria* (*S. tournefortii* Benth, *S. pinnatifida* subsp. *alpina* (Boiss.) Rech.f., *S. pinnatifida* subsp. *viridis* (Bornm.) Rech.f., *S. pinnatifida* subsp. *mucida* (Stapf) Rech.f., *S. tomentosa* Bertol) [49]. Collectively, these observations show that in-depth investigations of the chemical composition of volatile oils and hydrosols from individual plant organs of *Scutellaria* species remain scarce. Temporal variation in the relative abundances of specific secondary metabolites has been documented for *S. barbata* D.Don, collected in southern Alabama during June and July [47], and geographical differences in the volatile oil composition of *S. tournefortii* have been described for populations from the Golestan and Mazandaran provinces of Iran [49]. Hierarchical cluster analysis (HCA) identified distinct patterns of chemical similarity among the analyzed samples, particularly with respect to extraction products (volatile oil or hydrosol), species, and plant organ. However, these clustering patterns should be interpreted with caution and require further validation. Future research should include a broader range of *Scutellaria* species, increased biological replication, and additional multivariate statistical approaches, such as principal component analysis (PCA) and k-means clustering, to provide a more comprehensive evaluation of the relationships between volatile oils and hydrosols. Additional studies are required to confirm the interannual variation observed in *S. baicalensis* collected from the same population over two consecutive years and to evaluate the effects of plant material processing by comparing fresh and dried organs. Likewise, the differences observed in *S. supina* remain preliminary until validated through repeated multi-year sampling. A recent chemotaxonomic study employing hierarchical clustering, PCA, and k-means analyses of ten *Thymus* L. species from 18 countries revealed that essential oil composition clustered primarily by species rather than geographic origin [50]. Similarly, comprehensive chemotaxonomic analyses could provide valuable insights into species relationships and patterns of chemical variation within the genus *Scutellaria*.

### 4.2. Volatile Oils and Hydrosols

Several compounds were identified exclusively in hydrosols; for example, α-terpineol, geraniol, tridecane, and 2-methoxy-4-vinylphenol were identified in hydrosols obtained from *S. supina*, whereas benzaldehyde, tridecane, and eugenol were identified in hydrosols obtained from *S. baicalensis*. In *S. altissima*, benzaldehyde and eugenol were also identified exclusively in hydrosols. Furthermore, it has been reported that hydrosols obtained by hydrodistillation of rose flowers can be collected and subsequently redistilled to achieve complete recovery of volatile constituents. The differences in chemical composition observed between volatile oil samples and hydrosols may be attributed to the physicochemical properties of secondary metabolites or to physical processes occurring during hydrodistillation.

During boiling, plant cells absorb water, increasing internal pressure and rupturing cell walls, thereby facilitating the diffusion of volatile oils into the surrounding liquid. Steam distillation entails the simultaneous volatilization of water and aromatic compounds, followed by condensation of the vapor mixture. The volatility of oil constituents is determined by their vapor pressures and solubility in the aqueous phase rather than by their vaporization rates. As a result, compounds with higher water solubility distill before those with lower solubility. Upon condensation, differences in density and solubility cause phase separation, yielding two distinct layers: a volatile oil layer and the aqueous fraction of the distillate [28,29]. The aqueous fraction represents the hydrophilic portion of volatile compounds solubilized in the distillation water [22]. Given the complexity of these processes, further research is needed to elucidate the mechanisms underlying the observed compositional differences between volatile oils and hydrosols. The findings of this study suggest that hydrosols contain additional water-soluble volatile constituents that are absent or present only in trace amounts in the corresponding volatile oils. Therefore, characterizing both volatile oils and hydrosols provides a more comprehensive representation of the volatile metabolite profile of *Scutellaria* species. Hydrosols may therefore serve as a valuable complementary source of chemotaxonomic information and bioactive compounds and should be included alongside volatile oils in phytochemical studies.

### 4.3. Difference in Volatile Constituents Between Plant Organs

The observed differences in the relative abundances and occurrence of volatile constituents among *Scutellaria* plant organs warrant further investigation. This variation may be related to organ-specific differences in the biosynthesis, secretion, and accumulation of volatile compounds. Plants possess specialized anatomical structures for secretion and storage of volatile oils, including idioblasts, secretory cavities, secretory ducts, and glandular trichomes [28,42]. Furthermore, the biosynthesis and accumulation of secondary metabolites are strongly influenced by the plant’s developmental stage, the degree of tissue and organ differentiation, specific phenological phases, and fluctuations in abiotic factors throughout the growing season [51]. Secondary metabolites, including volatile compounds, play essential roles in plant adaptation, acting as defenses against microorganisms and herbivores [52]. At the same time, certain classes of compounds attract pollinators or fruit-dispersal agents or act as intraspecific and interspecific signaling molecules [43]. Plants can perceive biotic stimuli and respond dynamically to environmental challenges, including attacks by phytophagous insects, through complex chemical communication mechanisms. In response to herbivore attack, many plant species release volatile organic compounds (VOCs) into the atmosphere. These compounds function as stress signals, allowing neighboring plants to perceive the attack and pre-emptively activate their own defense responses, including the biosynthesis of metabolites that reduce leaf attractiveness or palatability to herbivorous insects [28]. Volatile organic compounds (VOCs) have been investigated in only a limited number of *Scutellaria* species. Analysis of VOCs emitted by fresh flowers of *S. baicalensis* grown under greenhouse and field conditions, as well as flowers obtained from different commercial suppliers, revealed substantial variation in chemical profiles, accompanied by differences in floral aroma. A total of 64 volatile constituents were identified, with sesquiterpene hydrocarbons representing the predominant class (55.3–81.4%) [53]. Similarly, the analysis of floral VOCs in *S. californica* A. Gray identified 51 compounds, of which sesquiterpene hydrocarbons accounted for 79.1% of the volatile fraction [54]. Organ-specific differences in VOC composition have been reported in *S. altissima;* 37 volatile constituents were identified in leaves, where non-terpene derivatives predominated (71.04%), whereas flowers contained only 11 volatile compounds, which were almost exclusively represented by monoterpene hydrocarbons (99.73%) [44]. By contrast, *S. brevibracteata* subsp. *subvelutina* (Rech.f.) Greuter & Burdet contained 27 volatile constituents in leaves and 40 in flowers, with sesquiterpene hydrocarbons representing the dominant chemical class in both organs (66.27% and 88.65%, respectively) [55]. Several VOCs previously reported in *Scutellaria*, including linalool, α-cubebene, β-bourbonene, β-caryophyllene, germacrene D, aromadendrene, 8-cadinene, and other compounds, were also identified in the volatile oils of the three *Scutellaria* species investigated in the present study. The presence of these compounds in both VOCs and volatile oils suggests they are common constituents of the volatile metabolome. Nevertheless, the relationship between VOC emissions and the composition of volatile oils in *Scutellaria* species remains poorly understood, particularly regarding organ-specific patterns and species-specific variations. Future investigations combining VOC profiling with volatile oil analysis under standardized experimental conditions would contribute to a better understanding of the ecological, physiological, and biochemical significance of these volatile constituents. The marked differences in volatile composition among vegetative and reproductive organs reported in previous studies are generally considered to reflect their distinct ecological roles. Constituents produced by leaves are primarily associated with plant defense against herbivores and pathogens through antifeedant, acaricidal, fumigant, and larvicidal activities, whereas floral constituents play an essential role in reproductive processes by mediating interactions with pollinators and other beneficial organisms [56]. Although some secondary metabolites are known to contribute to pollinator attraction, fruit dispersal, and plant communication [43], these ecological functions cannot be directly extrapolated to the genus *Scutellaria.* Current knowledge of pollination biology in this genus remains limited, and available studies indicate that pollinator interactions are highly species-specific and influenced by floral morphology, nectar characteristics, and flowering biology in addition to floral scent [57,58,59,60].

The micromorphological characteristics of the indumentum of different plant organs may also contribute to the observed variation in chemical composition. For example, micromorphological analyses of three *Scutellaria* species (*S. cypria* var. *cypria* Rechinger, *S. cypria* var. *elatior* Meikle, and *S. sibthorpii* (Benth.) Halácsy) revealed the presence of three types of capitate glandular trichomes that differed in the morphology of their secretory heads and the types of biologically active compounds they secreted [61]. Similarly, micromorphological analyses of different organs (stem, leaves, bracts, calyx, and corolla) of *S. altissima* revealed both non-glandular and glandular trichomes, with distinct morphology and distribution between vegetative and reproductive organs [44]. Furthermore, histochemical investigations of the indumentum (vegetative and reproductive organs) of *S. caucasica* [56] and *S. brevibracteata* subsp. *subvelutina* [55] demonstrated organ-specific differences in the accumulation of secondary metabolites associated with specific types of trichomes. Therefore, the organ-specific variation observed in the present study should not be interpreted solely in terms of ecological function, as it may also reflect differences in the distribution and density of secretory structures, tissue differentiation, developmental stage, and environmental factors influencing secondary metabolism. Consequently, the ecological and biological significance of the organ-specific volatile profiles observed in the present study remains to be elucidated. Future interdisciplinary studies integrating phytochemical, ecological, histoanatomical, histochemical, and molecular approaches will be essential to clarify the mechanisms underlying these organ-specific differences and the functional roles of volatile constituents in *Scutellaria*.

### 4.4. Potential Applications of Volatile Oils and Hydrosols

Secondary metabolites found in volatile oils demonstrate diverse biological activities, including anticancer and antiparasitic effects [62]. These compounds also exhibit antiviral properties, particularly against herpes simplex virus (HSV), influenza virus (INF), Junin virus (JUNV), human immunodeficiency virus (HIV), and yellow fever virus (YFV). When applied topically, volatile oils can alleviate pain and inflammation and reduce the severity of local clinical symptoms. Their antibacterial properties further decrease the risk of bacterial superinfection [63]. Compounds with antibacterial activity include aldehydes, alcohols, phenols, ketones, esters, oxides, epoxides, methyl ethers, and terpene hydrocarbons. Among these, aldehydes, alcohols, and phenols are generally regarded as the most potent antimicrobial agents. Additionally, volatile oils can modulate microbial virulence factors, thereby diminishing microbial pathogenicity [64]. Volatile oils extracted from various taxa of the genus *Scutellaria*, including *S. barbata* [65], *S. sieberia* Benth., *S. rupestris* Boiss. et Heldr. ssp. *adenotricha* (Boiss. et Heldr.) Greuter et Burdet [66], *S. sibthorpii*, *S. cypria* var. *cypria*, *S. cypria* var. *elatior* [62], *S. grossa* Wall. ex Benth. [26], *S. araxensis* [67], *S. condensata* subsp. *pycnotricha* [68] and *S. oxystegia* Grossh [69], as well as volatile oil and the compounds linalool and trans-nerolidol isolated from *S. albida* ssp. *albida* [70] have demonstrated antibacterial activity against certain Gram-positive and Gram-negative bacteria. Volatile oils represent potential natural sources of valuable compounds with broad applications in the food, cosmetic, and pharmaceutical industries. However, most available data are derived from *in vitro* studies, and robust clinical evidence supporting the efficacy of these volatile oils *in vivo* remains limited [28]. Volatile oils obtained from *Scutellaria* species demonstrate potential for application in organic agriculture. One study evaluated the allelopathic activity of volatile oil extracted from the aerial parts of *S. strigillosa* Hemsley, including flowers and young fruits, against *Amaranthus retroflexus* L. and *Poa annua* L. The volatile oil inhibited the development of both species, with *A. retroflexus* exhibiting greater sensitivity. At a concentration of 1 µL/mL, root elongation was reduced by 86.6%, and at 3 µL/mL, plant growth was completely inhibited [71]. Furthermore, hydrosols from certain Lamiaceae taxa represent promising natural alternatives for pest control in eco-agrosystems, either individually or in combination [72]. Additional research is necessary to investigate the potential applications of volatile oils and hydrosols from *Scutellaria* species, including the three species analyzed in this study. Volatile oils and hydrosols could also be used in interdisciplinary studies targeting the taxonomy of the genus *Scutellaria*. Approaching the taxonomy of this genus through interdisciplinary studies using multiple independent datasets from geographical, morphological, biochemical, cytological, and molecular sources has proven very useful [6].

### 4.5. Potential Sources of Diisobutyl Phthalate (DiBP) Contamination

Contaminants of synthetic origin are occasionally detected in volatile oils and plant extracts and may be mistaken for naturally occurring plant metabolites. Among these contaminants, phthalate esters have been reported [73]. Phthalates are synthetic compounds widely used as plasticizers to improve the flexibility and durability of polymeric materials and are recognized as environmental contaminants with potential reproductive and endocrine-disrupting effects [74]. Some studies suggest that plants are capable of absorbing and accumulating phthalates from a contaminated environment; for example, diisobutyl phthalate (DiBP) and phthalic acid have been reported in volatile oils obtained by hydrodistillation of the roots of the *Achillea tenuifolia* Lam., harvested from Iran, highlighting the importance of implementing rigorous quality control procedures during the production and analysis of medicinal plants [75]. Because phthalates can enter the human body through multiple exposure routes, including the terrestrial food chain, contamination of medicinal plants and herbal products has become an important issue for food and pharmaceutical safety [75,76]. Edible vegetable oils have likewise been recognized as a potential source of human exposure to phthalates [77]. Diisobutyl phthalate (DiBP) was detected in several samples of the three *Scutellaria* species analyzed in the present study. The origin of this compound cannot be established based on the available data. It may reflect environmental contamination of the plant material resulting from phthalate uptake during growth, or it may have been introduced during sample handling, extraction, storage, or instrumental analysis. Although all samples were processed using the same hydrodistillation apparatus and analyzed under identical GC-MS conditions, thereby reducing the likelihood of analytical contamination, this possibility cannot be completely excluded. Therefore, additional investigations, including procedural blanks, environmental analyses, and replicate sampling, are required to determine the origin of DiBP in the analyzed samples.

## 5. Conclusions

This study presents the first comprehensive characterization of volatile oils and their corresponding hydrosols obtained from four plant organs (stems, leaves, flowers, and fruits) of *S. altissima*, *S. baicalensis* and *S. supina* cultivated in the Republic of Moldova. The analyzed taxa biosynthesized and accumulated a chemically diverse spectrum of volatile constituents spanning eleven classes of secondary metabolites. Aldehydes, alkanes, and phenols were detected predominantly or at higher concentrations in hydrosols compared to the corresponding volatile oils. These findings indicate that hydrosols should not be regarded as mere by-products of hydrodistillation but as valuable sources of biologically relevant volatile compounds warranting inclusion in phytochemical, chemotaxonomic, and potential biological applications. A common chemical core comprising nine compounds (1-octen-3-ol, acetophenone, linalool, α-terpineol, geraniol, eugenol, E-nerolidol, hexahydrofarnesyl acetone, and palmitic acid) was identified across the analyzed *Scutellaria* species. Furthermore, 2-butanone, 4-(2,2-dimethyl-6-methylenecyclohexyl)-, 1-undecanol, a-(Z,E)-farnesene, a-patchoulene, n-hexyl salicylate, and myristic acid may represent potential chemotaxonomic markers and should be evaluated using a broader sampling of *Scutellaria* species and populations. Future research should investigate interannual, geographical, processing-method-related, and plant-organ-related variations in volatile composition using interdisciplinary approaches. These approaches should integrate metabolomic, micromorphological, histoanatomical, genetic, ecological, chemotaxonomic, and biochemical analyses, together with advanced statistical methods, across a broader range of *Scutellaria* species, populations, years, and geographical regions. Additional studies are needed to clarify the physicochemical mechanisms governing the partitioning of volatile compounds between volatile oils and hydrosols. Such research will enhance understanding of the phytochemistry, ecology, and chemotaxonomy of *Scutellaria* species. Furthermore, future investigations should determine the sources of diisobutyl phthalate (DiBP) contamination and further assess the biological activities and practical applications of volatile oils and hydrosols.

## Figures and Tables

**Table 1:** Secondary metabolite classes detected in volatile oil and hydrosol samples of *S. altissima*.

Classes of Secondary Metabolites	1 os	1 hs	1 ol	1 hl	1 of	1 hf	1 or	1 hr
Acids (AC)	14.53	ND	13.41	ND	16.76	ND	**21.68**	ND
Alcohols (AL)	4.87	10.57	**15.97**	11.22	3.32	9.36	2.30	0.50
Aldehydes (AD)	ND	0.47	ND	1.38	ND	**2.10**	ND	0.24
Esters (E)	2.11	ND	1.20	ND	**1.81**	ND	1.73	ND
Ketones (K)	2.83	0.96	7.83	1.49	**7.85**	0.58	4.77	0.26
Monoterpene hydrocarbons (MH)	0.10	ND	**0.12**	ND	ND	ND	ND	ND
Oxygenated monoterpenes (OM)	32.31	86.79	29.32	83.94	26.1	85.63	43.74	**97.04**
Oxygenated sesquiterpenes (OS)	1.01	ND	**2.47**	ND	0.83	ND	0.79	ND
Phenols (P)	ND	0.57	ND	0.44	ND	0.28	ND	**0.62**
Sesquiterpene hydrocarbons (SH)	0.44	ND	0.76	ND	0.43	ND	**0.80**	ND

Notes: Blue shading indicates metabolite classes detected in all hydrosol samples; yellow shading indicates metabolite classes detected in all volatile oil samples; green shading indicates metabolite classes detected in all samples. Bold values indicate the highest relative abundance. Abbreviations: o = volatile oil; h = hydrosol; l = leaves; s = stems; f = flowers; r = fruits; 1 = *S. altissima* plants harvested in 2023; ND = not detected.

**Table 2:** Secondary metabolite classes detected in volatile oil and hydrosol samples of *S. baicalensis* harvested in 2022.

Classes of Secondary Metabolites	2 os	2 hs	2 ol	2 hl	2 of	2 hf	2 or	2 hr
Acids (AC)	ND	**2.11**	0.99	0.76	0.22	0.33	1.05	0.71
Alcohols (AL)	4.83	37.96	22.11	51.98	3.27	**65.19**	5.06	53.99
Aldehydes (AD)	ND	**2.26**	ND	0.46	ND	0.34	ND	1.13
Alkanes (AK)	ND	**0.98**	ND	0.51	ND	ND	ND	0.31
Ketones (K)	ND	3.49	0.67	14.99	0.94	1.66	2.20	**27.40**
Other (O)	0.86	ND	0.54	ND	1.57	ND	**1.86**	ND
Oxygenated monoterpenes (OM)	1.85	**24.80**	3.63	3.09	0.24	19.26	0.45	4.50
Oxygenated sesquiterpenes (OS)	19.27	8.15	10.67	6.88	23.27	1.44	**28.52**	4.85
Phenols (P)	ND	**2.87**	ND	2.84	ND	2.38	ND	2.72
Sesquiterpenes Hydrocarbons (SH)	69.84	15.32	61.36	18.01	**70.15**	9.42	59.85	4.05

Notes: Blue shading indicates metabolite classes detected in all hydrosol samples; yellow shading indicates metabolite classes detected in all volatile oil samples; green shading indicates metabolite classes detected in all samples. Bold values indicate the highest relative abundance. Abbreviations: o = volatile oil; h = hydrosol; l = leaves; s = stems; f = flowers; r = fruits; 2 = S. *baicalensis* plants harvested in 2022; ND = not detected.

**Table 3:** Secondary metabolite classes detected in volatile oil and hydrosol samples of *S. baicalensis* harvested in 2023.

Classes of Secondary Metabolites	3 os ^1^	3 hs ^1^	3 os	3 hs	3 ol ^1^	3 hl ^1^	3 ol	3 hl	3 of	3 hf	3 or	3 hr
Acids (AC)	**27.18**	ND	ND	1.13	ND	ND	0.69	ND	ND	ND	1.69	ND
Alcohols (AL)	ND	23.95	1.95	**57.13**	0.06	2.35	4.09	50.49	ND	5.40	5.81	32.36
Aldehydes (AD)	ND	**4.73**	ND	ND	ND	2.02	ND	ND	ND	ND	ND	ND
Alkanes (AK)	ND	**9.80**	ND	2.62	ND	3.81	ND	2.33	ND	4.95	ND	0.92
Ketones (K)	1.75	15.19	1.22	6.39	0.21	40.53	0.30	2.50	5.31	**73.00**	4.99	51.85
Other (O)	ND	ND	0.73	ND	0.38		0.21	ND	1.33	ND	**2.57**	ND
Oxygenated monoterpenes (OM)	0.80	22.26	ND	9.25	0.41	**25.12**	0.29	14.31	ND	ND	ND	2.74
Oxygenated sesquiterpenes (OS)	10.20	ND	10.36	5.07	8.49	ND	4.79	ND	21.93	5.95	**31.76**	2.87
Phenols (P)	ND	**14.69**	ND	9.26	ND	11.11	ND	6.50	ND	5.59	ND	3.82
Sesquiterpene hydrocarbons (SH)	54.03	9.37	81.45	6.89	**90.46**	15.07	89.01	23.86	66.96	5.12	53.17	3.51

Notes: Blue shading indicates metabolite classes detected in all hydrosol samples; yellow shading indicates metabolite classes detected in all volatile oil samples; green shading indicates metabolite classes detected in all samples. Bold values indicate the highest relative abundance. Abbreviations: o = volatile oil; h = hydrosol; l = leaves; s = stems; f = flowers; r = fruits; s ^1^ = fresh stems; l ^1^ = fresh leaves; 3 = S. *baicalensis* plants harvested in 2023; ND = not detected.

**Table 4:** Secondary metabolite classes detected in volatile oil and hydrosol samples of *S. supina* harvested in 2022 and 2023.

Classes of Secondary Metabolites	4 os	4 hs	5 os	5 hs	4 ol	4 hl	5 ol	5 hl	5 of	5 hf	5 or	5 hr
Acids (AC)	**19.10**	ND	ND	0.90	ND	ND	ND	0.70	ND	2.41	ND	2.01
Alcohols (AL)	14.33	29.27	6.59	44.85	14.78	59.31	12.99	**71.10**	0.54	4.48	2.10	17.39
Alkanes (AK)	ND	1.00	ND	0.68	ND	0.92	ND	1.19	ND	2.35	ND	**2.40**
Ketones (K)	11.35	10.87	3.15	0.24	**14.22**		10.62		3.82	10.67	5.31	13.82
Oxygenated monoterpenes (OM)	21.95	**52.77**	4.96	36.79	7.37	30.36	2.60	11.11	0.20	9.60	0.37	5.86
Oxygenated sesquiterpenes (OS)	8.37	ND	9.70	3.44	**23.45**	ND	9.24	4.43	7.47	14.69	18.45	19.16
Phenols (P)	0.55	1.90	0.12	0.40	ND	**1.28**	0.27	0.81	ND	ND	ND	ND
Sesquiterpene hydrocarbons (SH)	18.96	4.19	44.73	5.15	37.76	8.13	37.04	5.43	**61.88**	14.92	15.75	6.46

Notes: Blue shading indicates metabolite classes detected in all hydrosol samples; yellow shading indicates metabolite classes detected in all volatile oil samples; green shading indicates metabolite classes detected in all samples. Bold values indicate the highest relative abundance. Abbreviations: o = volatile oil; h = hydrosol; l = leaves; s = stems; f = flowers; r = fruits; 4 = *S. supina* plants harvested in 2022 and 5 harvested in 2023; ND = not detected.

## Data Availability

Raw datasets generated and analyzed during this study are not available online because they are part of an ongoing study, which includes two other *Scutellaria* species, and are available from the corresponding author upon reasonable request.

## Appendix A

**Table A1:** Plant materials were used to extract volatile oils and hydrosols and to analyze the volatile constituents of the hydrosols.

Plant Species	Harvest Date	Plant Organ/Amount of Material (g) Subjected to Hydrodistillation	Volume of Hydrosol (mL) from Which Volatile Compounds Were Extracted
*S. altisima*	6 June 2023	Stems: 100; leaves: 100; flowers: 40.0696; fruits: 77.8051	Stems: 6; leaves: 6.5; flowers: 7.5; fruits: 7
*S. baicalensis*	22 August 2022	Stems: 100; leaves: 100; flowers: 20. 9267; fruits: 32.7625	Stems: 7.5; leaves: 7.5; flowers: 7; fruits: 7
	14 September 2023	Fresh stems: 100; dried stems: 100; fresh leaves: 100; dried leaves: 100; flowers: 8.8263; fruits: 61.1511	Fresh stems: 6; dried stems: 6; fresh leaves: 9.5; dried leaves: 8.5; flowers: 7; fruits: 6.5
*S. supina*	22 August 2022	Stems: 100; leaves: 100	Stems: 7.5; leaves: 7.5
	6 June 2023	Stems: 63.527; leaves: 76.2534; flowers: 26.5277; fruits: 14.3184	Stems: 6; frunze:6.5; flowers: 7.5; fruits: 7.5

**Table A2:** Chemical composition (%) of volatile oil and hydrosol samples from *S. altissima* harvested in 2023.

Compounds	CF	CSM	LRI	1 os	1 hs	1 ol	1 hl	1 of	1 hf	1 or	1 hr
Benzaldehyde	C_7_H_6_O	AD		ND	0.47	ND	1.38	ND	**2.10**	ND	0.24
1-Octen-3-ol	C_8_H_16_O	AL		1.55	9.08	2.94	**9.95**	1.22	8.51	ND	0.50
3-Octanol	C_8_H_18_O	AL		0.39	**1.49**	0.45	1.27	ND	0.85	ND	ND
D-limonene	C_10_H_16_	MH	1027	0.10	ND	0.12	ND	ND	ND	ND	ND
Acetophenone	C_8_H_8_O	K	1061	0.10	0.96	0.28	**1.49**	ND	0.58	ND	0.26
Linalool	C_10_H_18_O	MO	1097	24.71	59.21	20.79	55.36	20.24	**61.72**	29.96	60.41
α-Terpineol	C_10_H_18_O	MO	1233/1234 ^a^	2.74	13.58	3.30	13.77	2.46	11.44	5.56	**17.85**
Nerol	C_10_H_18_O	MO	1243	1.22	3.66	1.27	3.70	0.80	3.12	1.89	**4.73**
Geraniol	C_10_H_18_O	MO	1252	3.64	10.35	3.97	11.11	2.60	9.35	6.32	**14.05**
Eugenol	C_10_H_12_O_2_	P	1284	ND	0.57	ND	0.44	ND	0.28	ND	**0.62**
β-ionone	C_13_H_20_O	K	1440	0.46	ND	**1.38**	ND	0.39	ND	0.46	ND
α-patchoulene *	C_15_H_24_	SH	1465	0.44	ND	0.76	ND	0.43	ND	**0.80**	ND
E-nerolidol	C_15_H_26_O	SO	1481	1.01	ND	**2.47**	ND	0.83	ND	0.79	ND
n-hexyl salicylate	C_13_H_18_O_3_	E	1639	**2.11**	ND	1.20	ND	1.81	ND	1.73	ND
Myristic acid	C_14_H_28_O_2_	AC	1680	ND	ND	**2.46**	ND	1.04	ND	1.11	ND
Hexahydrofarnesyl acetone	C_18_H_36_O	K	1821	2.27	ND	4.94	ND	**6.80**	ND	3.97	ND
Diisobutyl phthalate	C_16_H_22_O_4_	E	1830/1829 ^a^	41.80	0.65 ^a^	28.92	1.53 ^a^	42.89	2.05	24.20	1.33
Farnesyl acetone	C_18_H_30_O	K	1855	ND	ND	**1.23**	ND	0.66	ND	0.34	ND
Palmitic acid	C_16_H_32_O_2_	AC	1882	14.53	ND	10.95	ND	15.72	ND	**20.56**	ND
Phytol	C_20_H_40_O	AL	2055	2.92	ND	**12.57**	ND	2.11	ND	2.30	ND
Relative abundance (%)				58.20	99.35	71.08	98.47	57.11	97.95	75.80	98.67
Number of identified compounds				15	9	17	9	14	9	13	8

Notes: Blue = secondary metabolites detected in all hydrosol samples; yellow = secondary metabolites detected in all volatile oil samples; green = secondary metabolites detected in all samples; Bold = highest relative abundance; * = tentatively identified compounds; ^a^ = different linear retention index. Abbreviations: CF = chemical formula; CSM = class of secondary metabolites; LRI = linear retention index; o = volatile oil; h = hydrosol; l = leaves; s = stems; f = flowers; r = fruits; 1 = *S. altissima* plants harvested in 2023; ND = not detected.

**Table A3:** Chemical composition (%) of volatile oil and hydrosol samples from *S. basicalensis* harvested in 2022.

Compounds	CF	CSM	LRI	2 os	2 hs	2 ol	2 hl	2 of	2 hf	2 or	2 hr
Benzaldehyde	C_7_H_6_O	AD		ND	**2.26**	ND	0.34	ND	0.46	ND	1.13
1-octen-3-ol	C_8_H_16_O	AL		0.85	24.54	18.52	**59.32**	2.86	48.44	4.03	50.22
3-octanol	C_8_H_18_O	AL		0.77	**11.79**	2.82	5.87	0.21	3.07	0.29	3.36
Acetophenone	C_8_H_8_O	K	1061	ND	2.37	ND	1.66	0.39	14.55	0.84	**27.00**
Linalool	C_10_H_18_O	MO	1099	1.63	**16.23**	2.79	12.46	0.24	3.09	0.33	3.63
α-Terpineol	C_10_H_18_O	MO	1232/1233 ^a^	0.22	3.38 ^a^	0.49	**4.70 ^a^**	ND	ND	0.12	ND
Carvone	C_10_H_14_O	MO	1250	ND	**2.91**	ND	ND	ND	ND	ND	0.46
Geraniol	C_10_H_18_O	MO	1236/1252 ^a^	ND	**2.28 ^a^**	0.35	2.10 ^a^	ND	ND	ND	0.41 ^a^
Tridecane	C_13_H_28_	AK	1260	ND	**0.98**	ND	ND	ND	0.51	ND	0.31
γ-Elemene	C_15_H_24_	SH	1279/1306 ^a^	ND	ND	0.93 ^a^	ND	**0.96**	ND	0.71	ND
α-cubebene	C_15_H_24_	SH	1284	**0.66**	ND	0.61	ND	0.37	ND	0.64	ND
Eugenol	C_10_H_12_O_2_	P	1284	ND	**2.87**	ND	2.38	ND	2.84	ND	2.72
α-copaene	C_15_H_24_	SH	1293/1342 ^a^	**1.66**	0.95	1.43 ^a^	ND	0.72	1.15	0.96	0.46
β-Bourbonene	C_15_H_24_	SH	1295	4.82	1.01	6.03	**8.06**	0.59	ND	4.80	0.68
β-Eiemene	C_15_H_24_	SH	1297	**1.40**	ND	ND	ND	0.41	ND	0.68	ND
β-Caryophyllene	C_15_H_24_	SH	1411/1415 ^a^/1414 ^b^	29.14	3.83	23.44 ^a^	ND	**37.99** ^b^	10.65	26.08 ^b^	1.50
β-Cubebene	C_15_H_24_	SH	1417	1.26	1.02	**2.35**	ND	1.69	ND	1.79	ND
α-Bergamotene	C_15_H_24_	SH	1417	**3.40**	ND	ND	ND	ND	ND	ND	ND
β-copaene	C_15_H_24_	SH	1427 ^a^/1424	**3.85 ^a^**	0.81	1.07	ND	0.68	ND	0.89	ND
(E)-β-Famesene	C_15_H_24_	SH	1430/1427 ^a^	**3.97**	1.06 ^a^	1.39	ND	1.02	ND	0.95	ND
α-Humulene	C_15_H_24_	SH	1431/1429 ^a^	1.04	0.94 ^a^	3.22	ND	**4.06**	1.06 ^a^	2.99	ND
Aromandendrene	C_15_H_24_	SH	1433	**3.21**	ND	1.24	ND	0.76	ND	0.95	ND
1-Undecanol	C_11_H_24_O	AL	1438	**3.21**	**0.95**	ND	ND	ND	ND	ND	ND
β-Ionone	C_13_H_20_O	K	1440	ND	**0.61**	ND	ND	ND	ND	ND	ND
Germacrene D	C_15_H_24_	SH	1445 ^a^/1442	12.03 ^a^	1.73	6.83	0.56	**14.35**	2.17	7.23	0.61
α-(Z,E)-Farnesene	C_15_H_24_	SH	1445	ND	ND	**7.95**	0.36	ND	0.70	5.88	0.41
Bicyclogermacrene	C_15_H_24_	SH	1449	ND	ND	0.58	ND	**0.73**	0.44	0.60	ND
α-Muurolene	C_15_H_24_	SH	1451	ND	ND	**1.29**	ND	1.09	1.03	1.01	ND
Farnesene	C_15_H_24_	SH	1453	ND	0.54	0.86	ND	**2.41**	ND	0.59	ND
γ-cadinene	C_15_H_24_	SH	1456	**1.87**	0.88	0.91	0.27	1.52	0.81	1.61	0.39
δ-Cadinene	C_15_H_24_	SH	1460/1459^a^	1.53	**2.55** ^a^	1.23	0.17 ^a^	0.80	ND	1.49	ND
*Neidentificat*	C_15_H_24_O	O	1478	0.86	ND	0.54	ND	1.57	ND	**1.86**	ND
E-Nerolidol	C_15_H_26_O	SO	1482	0.80	ND	0.56	ND	0.50	ND	**1.02**	ND
Spathulenol	C_15_H_24_O	SO	1489/1491 ^a^	1.79	1.74	1.71 ^a^	0.45	**2.19**	1.84	1.30	1.38
Caryophyllene oxide	C_15_H_24_O	SO	1492/1494 ^a^	11.62	4.76	5.58 ^a^	0.37	16.54	3.51	**20.27**	2.12
Humulene epoxide II	C_15_H_24_O	SO	1606	1.65	ND	ND	ND	1.63	ND	**1.77**	ND
Alloaromadendrene oxide-(1)	C_15_H_26_O	SO	1629	2.11	1.65	1.71	0.62	1.65	1.53	**2.81**	1.35
Cubenol	C_15_H_26_O	SO	1635	1.30	ND	1.11	ND	0.76	ND	**1.35**	ND
Hexahydrofarnesyl acetone	C_18_H_36_O	K	1821	ND	0.51	0.67	ND	0.55	0.44	**1.36**	0.40
Diisobutyl phthalate	C_16_H_22_O_4_	E	1728 ^a^/1730	3.37 ^a^	2.06	ND	ND	0.35	0.49	1.00	0.32
Palmitic acid	C_16_H_32_O_2_	AC	1880	ND	**2.11**	0.99	0.33	0.22	0.76	1.05	0.71
Phytol	C_20_H_40_O	AL	2055	ND	0.68	**0.77**	ND	0.20	0.47	0.74	0.41
Relative abundance (%)				96.63	97.94	100	100	99.65	99.51	99.00	99.68
Number of identified compounds				26	29	31	17	31	21	33	21

Notes: Blue = secondary metabolites detected in all hydrosol samples; yellow = secondary metabolites detected in all volatile oil samples; green = secondary metabolites detected in all samples; Bold = highest relative abundance; ^a,b^ = different linear retention index. Abbreviations: CF = chemical formula; CSM = class of secondary metabolites; LRI = linear retention index; o = volatile oil; h = hydrosol; l = leaves; s = stems; f = flowers; r = fruits; 2 = *S. baicalensis* plants harvested in 2022; ND = not detected.

**Table A4:** Chemical composition (%) of volatile oil and hydrosol samples from *S. basicalensis* harvested in 2023.

Compounds	CF	CSM	LRI	3 os ^1^	3 hs ^1^	3 os	3 hs	3 ol ^1^	3 hl ^1^	3 ol	3 hl	3 of	3 hf	3 or	3 hr
Benzaldehyde	C_7_H_6_O	AD		ND	**4.73**	ND	ND	ND	2.02	ND	ND	ND	ND	ND	1.93
1-octen-3-ol	C_8_H_16_O	AL		ND	14.99	1.39	42.57	0.04	2.35	2.79	**46.16**	ND	5.40	4.43	30.21
3-octanol	C_8_H_18_O	AL		ND	8.96	0.56	**14.56**	0.02	ND	0.47	4.33	ND	ND	0.31	2.15
Acetophenone	C_8_H_8_O	K	1061	ND	15.19	ND	6.39	0.21	40.53	0.01	2.50	0.26	**73.00**	1.59	51.85
Linalool	C_10_H_18_O	MO	1099/1097 ^a^	0.56	**16.05**	ND	5.89	0.10 ^a^	3.89	0.29 ^a^	8.49	ND	ND	ND	1.40
α-terpineol	C_10_H_18_O	MO	1232 ^a^/1233	0.24	6.21	ND	2.15	0.31 ^a^	**19.54**	ND	4.09	ND	ND	ND	ND
Geraniol	C_10_H_18_O	MO	1252	ND	ND	ND	1.21	ND	1.69	ND	**1.73**	ND	ND	ND	1.34
Tridecane	C_13_H_28_	AK	1260	ND	**9.80**	ND	2.62	ND	3.81	ND	2.33	ND	4.95	ND	0.92
γ-elemene	C_15_H_24_	SH	1279	0.59	ND	0.91	ND	**1.90**	ND	1.76	ND	1.07	ND	1.88	ND
α-cubebene	C_15_H_24_	SH	1284/1285 ^a^	0.48 ^a^	ND	0.83	ND	0.29 ^a^	ND	0.48	ND	0.19	ND	**0.98**	ND
Eugenol	C_10_H_12_O_2_	P	1284	ND	**14.69**	ND	9.26	ND	11.11	ND	6.50	ND	5.59	ND	3.82
α-copaene	C_15_H_24_	SH	1293	0.97	ND	1.29	ND	**1.75**	ND	1.63	ND	0.56	ND	ND	0.45
β-bourbonene	C_15_H_24_	SH	1295	5.39	ND	6.62	ND	4.16	11.50	4.29	**20.44**	1.41	ND	ND	0.73
β-eiemene	C_15_H_24_	SH	1297	ND	ND	1.16	ND	**1.46**	ND	0.89	ND	0.58	ND	ND	ND
β-caryophyllene	C_15_H_24_	SH	1415 ^a^/1411 ^b^ 1414	23.96 ^a^	ND	**37.87**	ND	35.66	ND	35.33	ND	32.45	ND	ND	0.52 ^b^
β-cubebene	C_15_H_24_	SH	1417	1.74	ND	2.51	ND	3.47	ND	**3.51**	ND	1.76	ND	ND	ND
β-copaene	C_15_H_24_	SH	1424	0.92	ND	1.28	ND	1.40	ND	1.45	ND	0.80	ND	**1.94**	ND
(E)-β-famesene	C_15_H_24_	SH	1430/1427 ^a^	0.71 ^a^	ND	1.27	ND	1.85	ND	2.06	ND	1.10	ND	**2.37**	ND
α-humulene	C_15_H_24_	SH	1431/1429 ^a^	2.54 ^a^	ND	3.62	ND	4.85	ND	4.25	ND	3.86	ND	**6.41**	ND
Aromandendrene	C_15_H_24_	SH	1433/1432 ^a^	1.33 ^a^	ND	1.41	ND	1.70	ND	1.52	ND	0.98	ND	**2.33**	ND
Germacrene D	C_15_H_24_	SH	1442	10.00	9.37	15.19	2.40	21.51	3.57	**22.42**	2.33	14.65	5.12	13.94	0.43
α-(Z,E)-Farnesene	C_15_H_24_	SH	1446/1445 ^a^	ND	ND	ND	ND	ND	ND	ND	ND	ND	ND	**7.19**	0.92 ^a^
Bicyclogermacrene	C_15_H_24_	SH	1449	0.82	ND	1.09	ND	1.08	ND	1.09	ND	1.06	ND	**2.59**	ND
α-Muurolene	C_15_H_24_	SH	1451	ND	ND	0.75	ND	2.37	ND	2.02	ND	1.31	ND	**2.42**	ND
Farnesene	C_15_H_24_	SH	1453	1.55	ND	1.64	ND	**2.96**	ND	2.40	ND	1.07	ND	1.84	ND
γ-Cadinene	C_15_H_24_	SH	1456	2.32	ND	3.29	2.64	2.92	ND	2.76	1.09	2.96	ND	**6.59**	0.46
δ-Cadinene	C_15_H_24_	SH	1460/1459 ^a^	0.71	ND	3.29	1.85 ^a^	1.13	ND	1.15	ND	1.15	ND	**2.69**	ND
*Neidentificat*	C_15_H_24_O		1478	ND	ND	0.72	ND	0.38	ND	0.21	ND	1.33	ND	**2.57**	ND
E-Nerolidol	C_15_H_26_O	SO	1482	ND	ND	0.73	ND	0.95	ND	0.41	ND	1.13	ND	**1.88**	ND
Spathulenol	C_15_H_24_O	SO	1489/1491 ^a^	1.02 ^a^	ND	0.55	1.33	1.43	ND	0.82	ND	3.02	ND	**11.21**	0.90
Caryophyllene oxide	C_15_H_24_O	SO	1492/1494 ^a^	4.89 ^a^	ND	1.20	2.11	2.97	ND	1.63	ND	13.58	5.95	**15.92**	1.01
Humulene epoxide II	C_15_H_24_O	SO	1606	ND	ND	ND	ND	ND	ND	ND	ND	1.72	ND	**2.75**	ND
Alloaromadendrene oxide-(1)	C_15_H_26_O	SO	1629	**2.58**	ND	1.08	1.63	1.73	ND	1.22	ND	1.63	ND	ND	0.96
Cubenol	C_15_H_26_O	SO	1635	**1.71**	ND	0.84	ND	1.41	ND	0.71	ND	0.85	ND	ND	ND
Hexahydrofarnesyl acetone	C_18_H_36_O	K	1821	1.75	ND	1.22	ND	ND	ND	0.29	ND	**5.05**	ND	3.40	ND
Diisobutyl phthalate	C_16_H_22_O_4_	E	1730	6.04	ND	4.29	2.26	ND	ND	0.63	ND	4.48	ND	ND	ND
Palmitic acid	C_16_H_32_O_2_	AC	1880	**27.18**	ND	ND	1.13	ND	ND	0.69	ND	ND	ND	1.69	ND
Phytol	C_20_H_40_O	AL	2055	ND	ND	ND	ND	ND	ND	0.83	ND	ND	ND	**1.07**	ND
Relative abundance (%)				93.96	100	95.71	97.74	100	100	99.37	100	95.52	100	100	100
Number of identified compounds				23	9	26	15	28	10	30	11	26	6	24	17

Notes: Blue = secondary metabolites detected in all hydrosol samples; yellow = secondary metabolites detected in all volatile oil samples; green = secondary metabolites detected in all samples; Bold = highest relative abundance; ^a,b^ = different linear retention index. Abbreviations: CF = chemical formula; CSM = class of secondary metabolites; LRI = linear retention index; o = volatile oil; h = hydrosol; l = leaves; s = stems; f = flowers; r = fruits; s ^1^ = fresh stems; l ^1^ = fresh leaves; 3 = *S. baicalensis* plants harvested in 2023; ND = not detected.

**Table A5:** Chemical composition (%) of volatile oil and hydrosol samples from *S. supina* harvested in 2022 and 2023.

Compounds	CF	CSM	LRI	4 os	4 hs	5 os	5 hs	4 ol	4 hl	5 ol	5 hl	5 of	5 hf	5 or	5 hr
1-Octen-3-ol	C_8_H_16_O	AL		10.87	29.27	4.34	44.85	14.78	59.31	10.57	**71.10**	ND	4.48	0.80	17.39
Acetophenone	C_8_H_8_O	K	1062	2.68	10.87	0.44	ND	ND	ND	ND	ND	0.21	9.18	0.71	**12.38**
Linalool	C_10_H_18_O	MO	1099/1062 ^a^	21.95	**45.49**	4.96	34.48	7.37	20.47	2.60	11.11	0.20 ^a^	7.20	0.37	5.86
α-terpineol	C_10_H_18_O	MO	1233	ND	3.19	ND	1.41	ND	**7.17**	ND	ND	ND	1.28		
Carvone	C_10_H_14_O	MO	1250	ND	**2.14**	ND	ND	ND	1.21	ND	ND	ND	ND	ND	ND
Geraniol	C_10_H_18_O	MO	1251	ND	**1.95**	ND	0.90	ND	1.51	ND	ND	ND	1.12	ND	ND
Tridecane	C_13_H_28_	AK	1260	ND	1.00	ND	0.68	ND	0.92	ND	1.19	ND	2.35	ND	**2.40**
2-Methoxy-4- vinylphenol *	C_9_H_10_O_2_	P	1271	ND	**1.90**	ND	0.40	ND	1.28	ND	0.81	ND	ND	ND	ND
γ-Elemene	C_15_H_24_	SH	1279	ND	ND	0.19	ND	ND	ND	0.46	ND	**1.60**	ND	ND	ND
α-Cubebene	C_15_H_24_	SH	1284/1479 ^a^	ND	2.18	0.66	2.07	ND	2.33	0.22	1.16	0.12 ^a^	**3.02**	0.42	2.12
Eugenol	C_10_H_12_O_2_	P	1284	**0.55**	ND	0.12	ND	ND	ND	0.27	ND	ND	ND	ND	ND
α-Copaene	C_15_H_24_	SH	1293	0.40	ND	0.79	ND	ND	ND	**1.59**	ND	1.43	0.93	1.14	ND
β-Bourbonene	C_15_H_24_	SH	1295	1.64	ND	2.04	ND	**6.86**	2.94	4.94	ND	0.81	ND	2.19	ND
β-Eiemene	C_15_H_24_	SH	1297/1296 ^a^	ND	ND	**1.52**	ND	ND	ND	1.08 ^a^	ND	0.69	ND	0.71	ND
2-butanone, 4-(2,2-dimethyl-6-methylenecyclohexyl) *	C_13_H_22_O	K	1298	ND	ND	ND	ND	ND	ND	0.46	ND	ND	ND	**1.52**	ND
β-Caryophyllene	C_15_H_24_	SH	1411	4.38	ND	**21.72**	1.43	8.54	ND	4.09	ND	7.85	1.52	1.85	ND
β-Cubebene	C_15_H_24_	SH	1416/1411 ^a^	ND	ND	1.36	ND		ND	2.22	ND	**4.18** ^a^	ND	1.01	ND
β-copaene	C_15_H_24_	SH	1424/1411 ^a^/1417 ^b^	4.62	ND	0.92	ND	**4.80** ^a^	ND	2.59	ND	1.84 ^b^	ND	0.80	ND
(E)-β-famesene	C_15_H_24_	SH	1427/1424 ^a^	1.53	ND	1.01	ND	**2.97**	ND	1.51	ND	2.75 ^a^	ND	0.80	ND
α-Humulene	C_15_H_24_	SH	1430	0.89	ND	**2.63**	ND	1.80	ND	ND	ND	ND	ND	ND	ND
Aromandendrene	C_15_H_24_	SH	1432	1.12	ND	0.72	ND	**3.24**	ND	2.24	ND	2.21	ND	1.14	ND
1-Undecanol	C_11_H_24_O	AL	1438/1432 ^a^	**3.45**	ND	2.24	ND	ND	ND	2.41	ND	0.54 ^a^	ND	1.30	ND
β-Ionone	C_13_H_20_O	K	1440/1438 ^a^	1.64	ND	0.45	ND	**4.74**	ND	3.67	ND	0.67 ^a^	ND	ND	ND
Germacrene D	C_15_H_24_	SH	1445/1442 ^a/^1443 ^b^/1440 ^c^	2.52 ^b^	0.89	8.72 ^a^	0.67	6.94 ^b^	1.57	12.72 ^b^	2.69	**33.16** ^c^	5.30	5.69 ^b^	2.61
α-muurolene	C_15_H_24_	SH	1450/1443 ^a^	0.36	ND	0.57	ND	ND	ND	0.87	ND	**2.07 ^a^**	1.33	ND	ND
γ-Cadinene	C_15_H_24_	SH	1455/1456 ^a^/1450 ^b^/ 1453 ^c^	0.78 ^c^	1.11	0.84	0.98	ND	1.29	0.91 ^a^	1.58	1.78 ^b^	**2.81**	ND	1.73
δ-Cadinene	C_15_H_24_	SH	1460/1461 ^a^/1453 ^b^	0.73	ND	1.02	ND	**2.61**	ND	1.61 ^a^	ND	1.40 ^b^	ND	ND	ND
E-Nerolidol	C_15_H_26_O	SO	1481	2.27	ND	2.03	ND	**3.10**	ND	ND	ND	ND	ND	ND	ND
Spathulenol	C_15_H_24_O	SO	1489	1.69	ND	3.36	1.38	9.69	ND	6.32	2.20	4.65	6.03	11.36	**11.83**
Caryophyllene oxide	C_15_H_24_O	SO	1492/1489 ^a^	3.25	ND	2.02	0.72	**5.42**	ND	ND	ND	1.16 ^a^	1.75	2.73	1.88
Humulene epoxide II	C_15_H_26_O	SO	1603/1492 ^a^	1.16	ND	2.29	0.64	**5.24**	ND	2.92	0.82	1.66 ^a^	2.22	4.36	3.19
Alloaromadendrene oxide-(1)	C_15_H_26_O	SO	1629	ND	ND	ND	0.70	ND	ND	ND	1.40	ND	**4.69**	ND	2.27
Hexahydrofarnesyl acetone	C_18_H_36_O	K	1821	7.03	ND	2.26	0.24	**9.48**	ND	6.49	ND	2.94	1.49	3.09	1.44
Diisobutyl phthalate	C_16_H_22_O_4_	E	1830/1821 ^a^	5.38	ND	30.75	7.54	2.42	ND	27.25	5.22	26.08 ^a^	40.88	58.02 ^a^	32.90
Palmitic acid	C_16_H_32_O_2_	AC	1880	**19.10**	ND	ND	0.90	ND	ND	ND	0.70	ND	2.41	ND	2.01
Relative abundance (%)				94.62	100	69.25	92.46	97.58	100	72.75	94.78	73.92	59.12	41.98	67.1
Number of identified compounds				23	11	26	16	16	11	23	11	22	18	19	13

Notes: Blue = secondary metabolites detected in all hydrosol samples; yellow = secondary metabolites detected in all volatile oil samples; green = secondary metabolites detected in all samples; Bold = highest relative abundance; * = tentatively identified compounds; ^a, b, c^ = different linear retention index. Abbreviations: CF = chemical formula; CSM = class of secondary metabolites; LRI = linear retention index; o = volatile oil; h = hydrosol; l = leaves; s = stems; f = flowers; r = fruits; 4 = *S. supina* plants harvested in 2022; 5 = *S. supina* plants harvested in 2023; ND =not detected.
